# Supplementary material for: Lipid-mediated intracellular delivery of recombinant bioPROTACs for the rapid degradation of undruggable proteins
Source: Nat Commun. 2024 Jul 10;15:5808. doi: 10.1038/s41467-024-50235-x (PMC11237011; doi:10.1038/s41467-024-50235-x)
Supplement: Supplementary file 1 — Supplementary Information [file 41467_2024_50235_MOESM1_ESM.pdf]

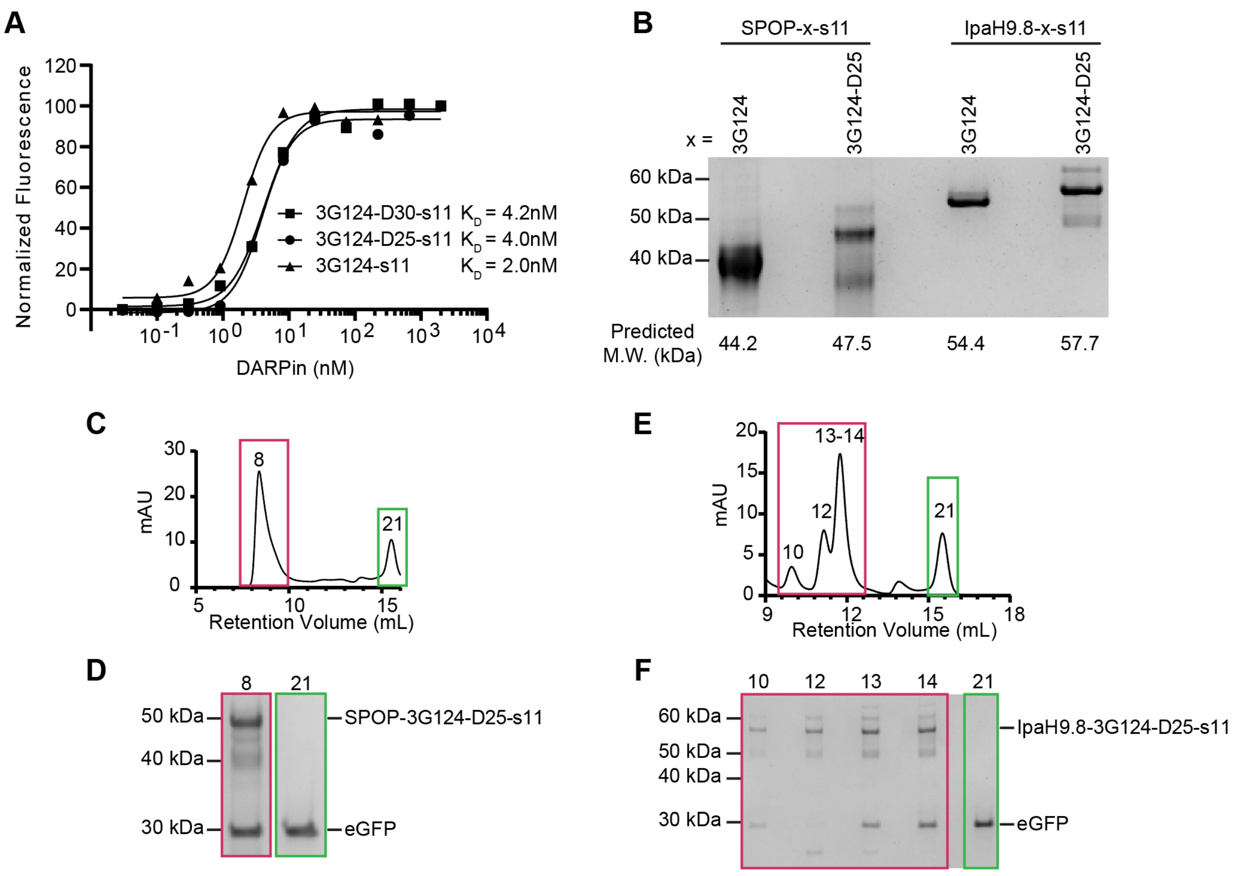

Supplementary Figure 1. Characterization of GFP-targeting bioPROTACs. **A.** Binding of 3G124 to GFP with or without ApP fusion. Data are mean of n = 2 wells. **B.** SDS-PAGE confirms that purified GFP bioPROTACs run at their predicted molecular weights. **C**. GFP and SPOP-3G124-D25-s11 incubated together and complexes were analyzed by analytical SEC to evaluate binding in solution. **D.** SDS-PAGE of primary peaks from C show co-elution of GFP with SPOP-based bioPROTAC. **E.** Chromatogram of GFP and IpaH9.8-3G124-D25-s11 complexes following incubation and SEC. **F.** SDS-PAGE of primary peaks from E show co-elution of GFP with IpaH9.8-based bioPROTAC. For C-F, the red box outlines bioPROTAC-containing fractions, while the green box indicates GFP-only fractions. Numbers above peaks and gel images in C-F correspond to the elution fraction number. Source data are provided as a Source Data file.

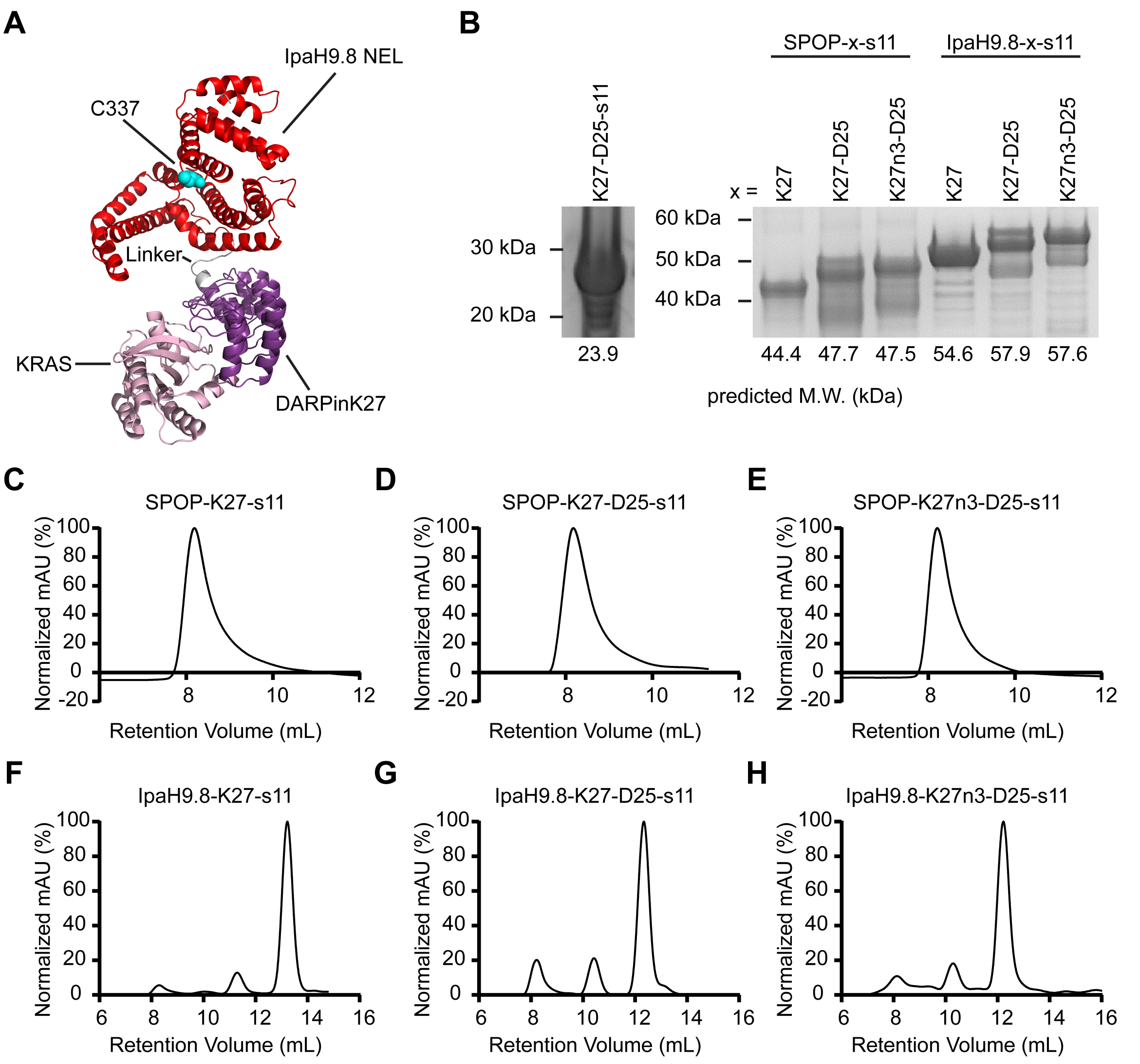

Supplementary Figure 2. Characterization of Ras-binding bioPROTACs. **A.** PyMol model of IpaH9.8-K27 bioPROTAC binding to KRAS. The catalytic cysteine residue responsible for target ubiquitination is highlighted in cyan. **B.** SDS-PAGE was performed to verify the successful purification of Ras-targeting bioPROTACs and control proteins. **C.-H.** Normalized chromatograms of SPOP- and IpaH9.8-fusion proteins. SPOP-fusions exhibit larger retention volumes compared to IpaH9.8 chimeras despite a lower monomeric molecular weight, indicating oligomerization. Source data are provided as a Source Data file.

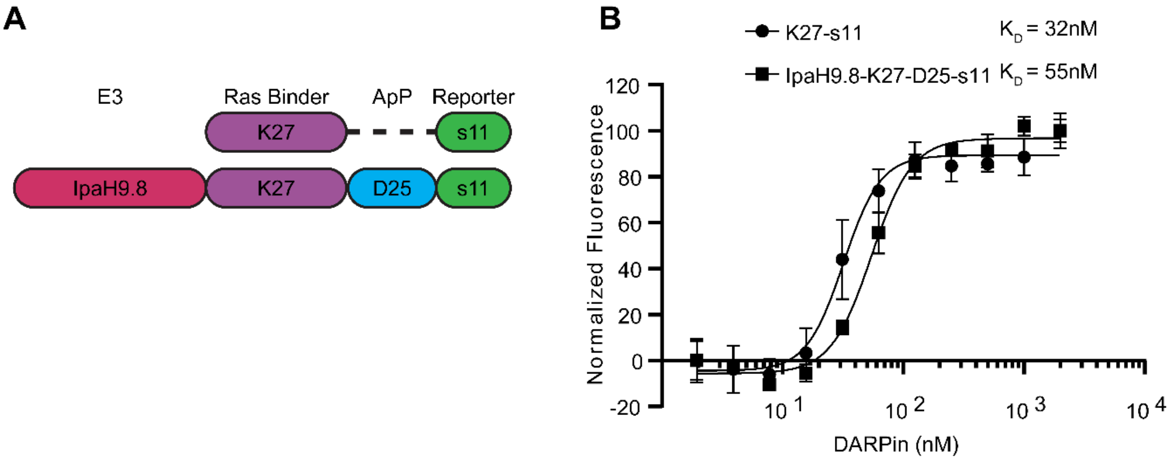

Supplementary Figure 3. Binding of purified Ras-targeting bioPROTACs to KRAS. **A.** Schematic of DARPinK27 with minimal modifications compared to the complete bioPROTAC with an N-terminal IpaH9.8 domain and a C-terminal D25 ApP. **B.** A Binding assay was performed, and the affinity of the complete anti-Ras bioPROTAC was found to be minimally affected by fusion to an E3 domain and a charged peptide sequence. Data are mean ± SD of n = 3 technical replicates. Source data are provided as a Source Data file.

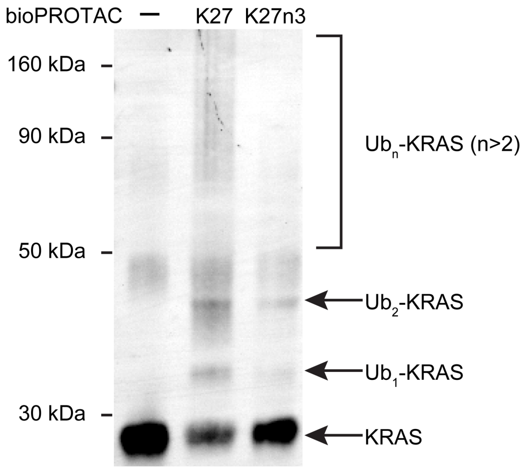

Supplementary Figure 4. *In vitro* ubiquitination assay. Purified bioPROTACs containing a binding domain (K27) or non-binding control (K27n3) were incubated with KRAS, E1, E2, and ubiquitin for 2 hours, and samples were analyzed by western blotting. Molecular weight bands above 24 kDa (recombinant KRAS) indicate ubiquitination (Ub) of KRAS. Source data are provided as a Source Data file.

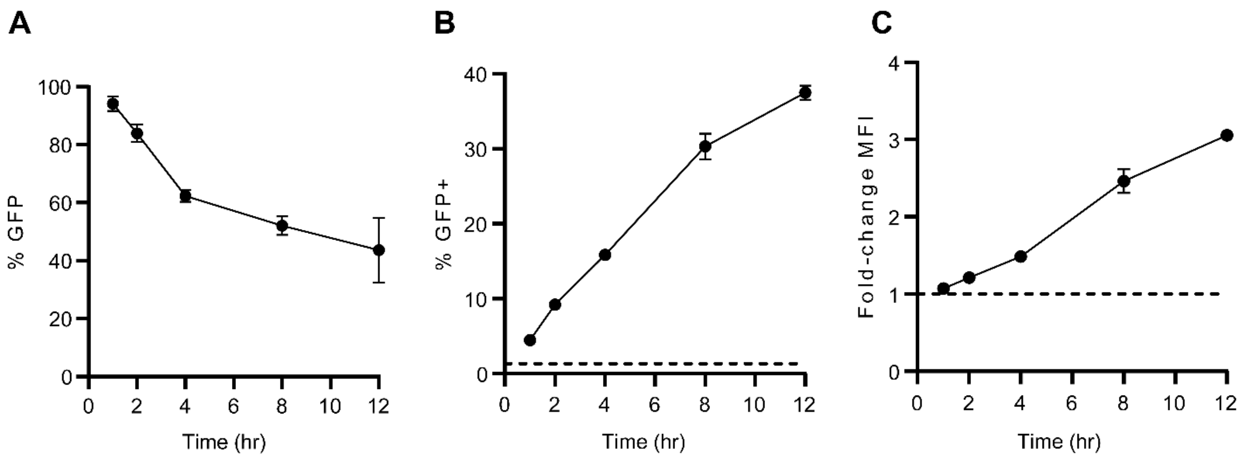

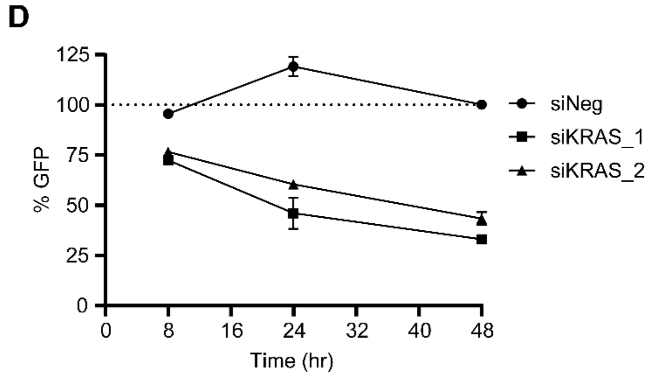

E

Cargo	Transfection Reagent	$t_{1/2}$(hr)
bioPROTAC	Lipofectamine 2000	9
siKRAS_1	Lipofectamine RNAiMAX	28
siKRAS_2	Lipofectamine RNAiMAX	38

Supplementary Figure 5. Comparison of bioPROTAC- and siRNA-mediated degradation rates. **A.** Ras-targeting bioPROTAC (500nM) was complexed with Lipofectamine 2000 and delivered into 293T GFP-KRAS cells. **B.** GFP-positive 293T GFP(1-10) cells following treatment with Lipofectamine:bioPROTAC. **C.** Fold-change MFI of 293T GFP(1-10) cells following treatment with Lipofectamine:bioPROTAC. **D.** Degradation in 293T GFP-KRAS cells transfected with a negative control siRNA or two anti-KRAS siRNA. **E.** Flow cytometry was performed up to 12 hours post-transfections and the degradation rate for each modality was estimated using a first-order decay equation. Data are mean ± SD of n = 3 separately transfected wells. Source data are provided as a Source Data file.

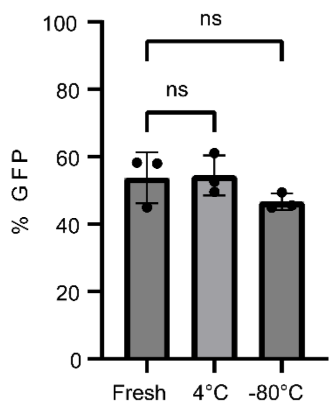

Supplementary Figure 6. Stability of purified proteins. Purified IpaH9.8-K27-D25-s11 was stored at either 4°C or -80°C for 28 days prior to Lipofectamine complexation. For both storage conditions, degradation efficiency was no different compared to newly-purified bioPROTAC. Data are mean ± SD of n = 3 biological replicates. Ordinary one-way ANOVA followed by multiple comparisons testing was performed. Source data are provided as a Source Data file.

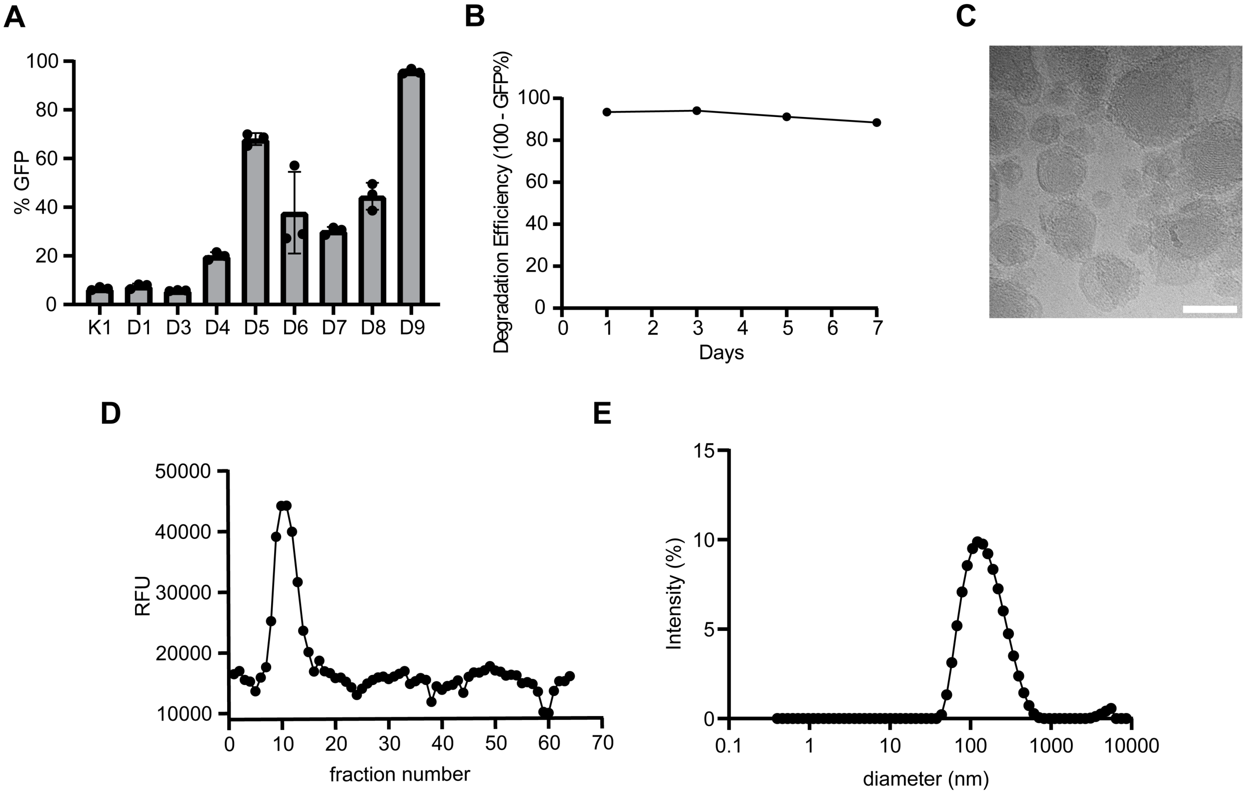

Supplementary Figure 7. Characterization of LNP:bioPROTAC formulations. **A.** Degradation efficiency of LNP library in 293T GFP-KRAS cells. **B.** The degradation efficiency of K1 was measured over the course of 1 week and did not exhibit a decline in activity. **C.** Cryo-TEM micrograph of K1:bioPROTAC LNPs. Scale bar = 100nm. **D.** The encapsulation efficiency of TAMRA-labeled bioPROTAC within K1 LNPs was determined by size exclusion column chromatography. **E.** Fractions corresponding to the major peak in D. were pooled and analyzed by dynamic light scattering to confirm LNP content. Data for A. are mean ± SD of n = 3 measurements. Source data are provided as a Source Data file.

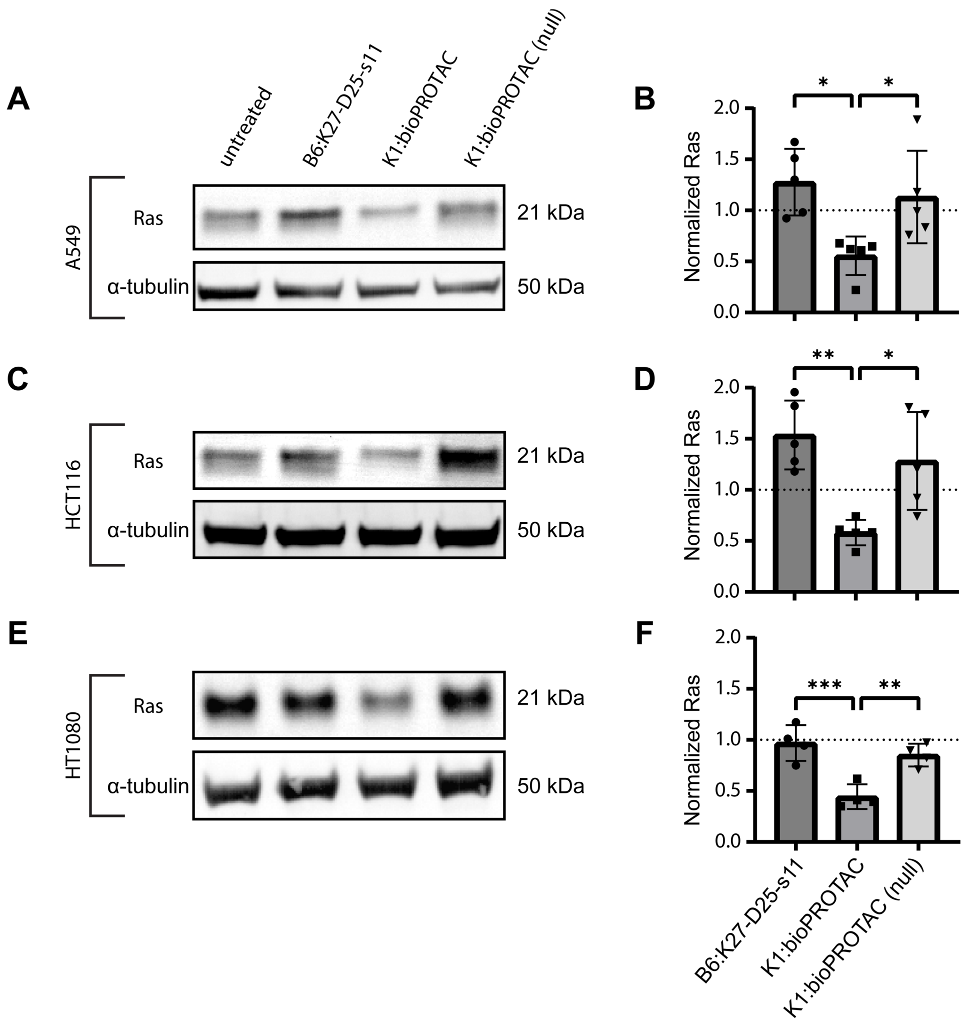

Supplementary Figure 8. Degradation of endogenous Ras in a panel of cancer cell lines. **A.** Representative western blot of A549 non-small cell lung cancer lysates after cells were treated with 100nM LNPs formulated either active bioPROTAC, a non-binding bioPROTAC, or an E3-deficient control. **B.** Band densitometry was performed on n = 5 biological replicates of A549 cells with the dotted line representing the normalized intensity of the untreated control. **C.** Same as A but with HCT116 colorectal cancer cells. **D.** Band densitometry was performed on n = 5 biological replicates of HCT116 cells. **E.** Same as A but with HT1080 fibrosarcoma cells. **F.** Band densitometry was performed on n = 4 biological replicates of HT1080 cells. Data mean ± SD. One-way ANOVA followed by multiple comparisons testing was performed. * $p \leq 0.05$, ** $p \leq 0.01$, *** $p \leq 0.001$. Source data are provided as a Source Data file.

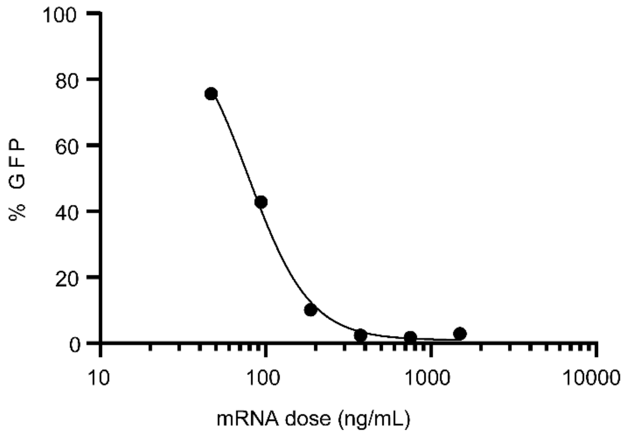

Supplementary Figure 9. Dose-dependent degradation in 293T GFP-KRAS cells treated with LNP:mRNA encoding the Ras-targeting bioPROTAC. Cells were treated for 8 hours and analyzed by flow cytometry. Data are mean of n = 2 technical replicates. Source data are provided as a Source Data file.

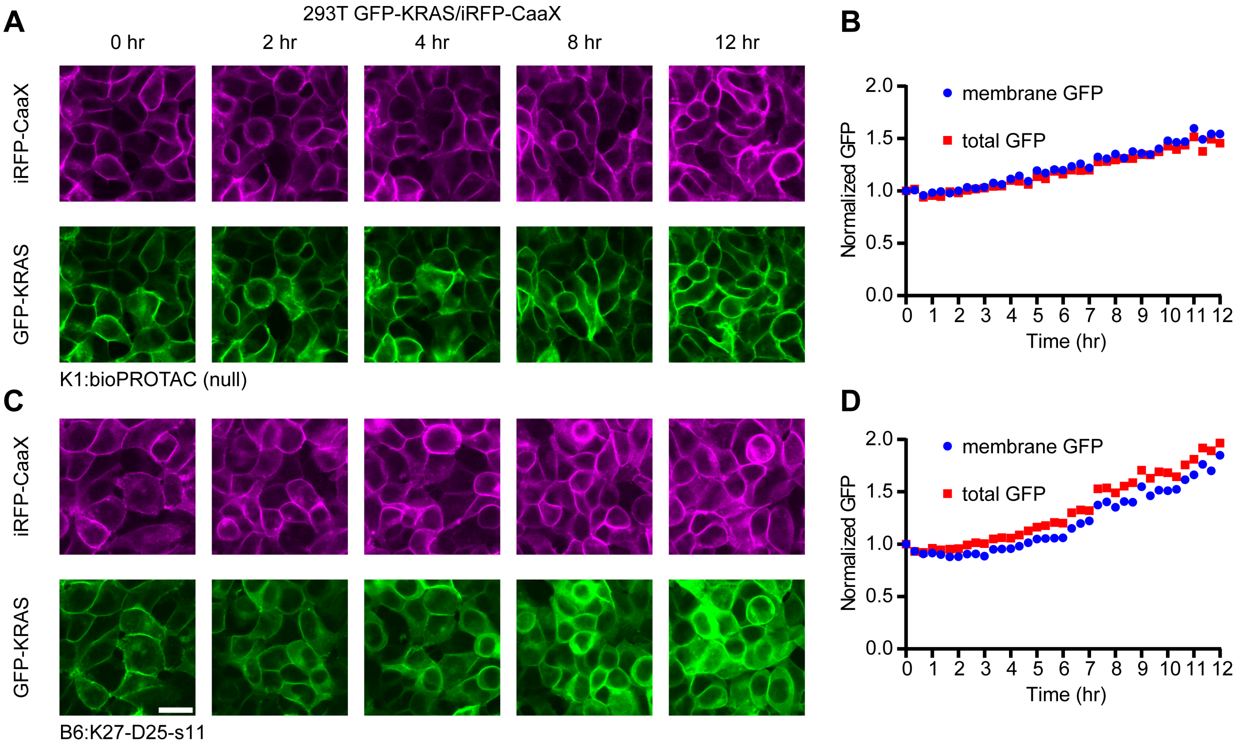

Supplementary Figure 10. Controls for degradation kinetics study. **A.** Dual reporting 293T GFP-KRAS/iRFP-CaaX cells were treated with K1:bioPROTAC(null) and monitored for 12 hours by fluorescence microscopy. **B.** Single-cell analysis of 400-500 cells at each time point following delivery of null bioPROTAC. **C.** Representative fluorescent images of reporter cells treated with the B6:K27-D25-s11. **D.** Single-cell analysis of 400-500 cells at each time point following delivery of K27-D25-s11. Scale bar applies to all microscopy images in A and C and is equal to 20μm. Source data are provided as a Source Data file.

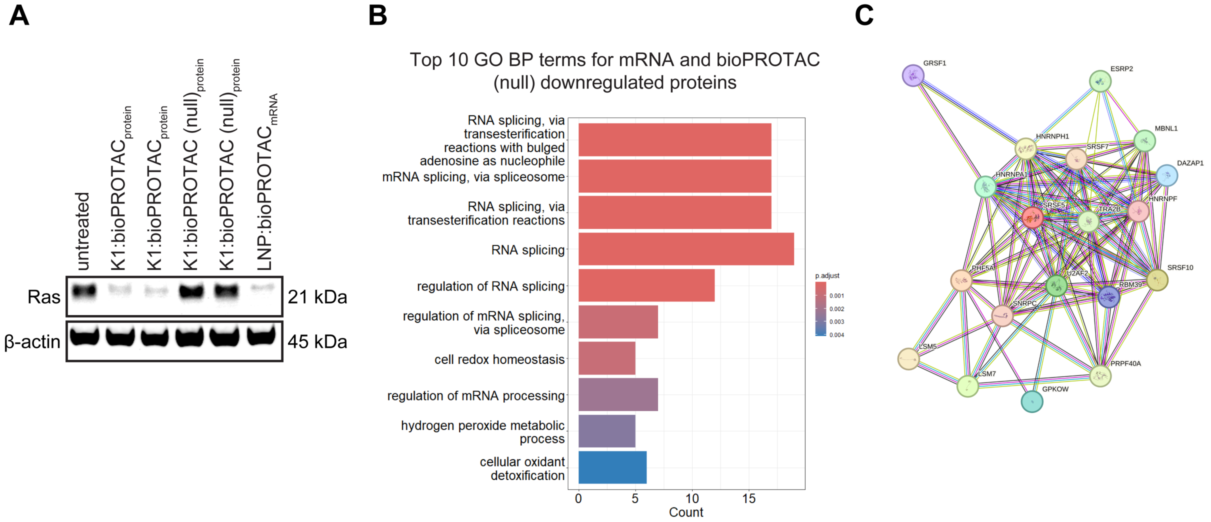

Supplementary Figure 11. Additional proteomics sample analyses. **A.** Immunoblotting of 293T extracts following protein (100nM) or mRNA (200ng/mL) delivery confirms expected Ras knockdown prior to MS analysis. Protein delivery samples were treated in duplicate. **B.** GO enrichment analysis was performed on the downregulated proteins shared between LNP:bioPROTAC$_{mRNA}$ and K1:bioPROTAC (null)$_{protein}$ treatment groups, and the top 10 terms by adjusted p-value were returned. **C.** STRING DB[1] interaction network for U2AF and related proteins associated with RNA splicing identified from pathway enrichment analysis. Source data are provided as a Source Data file.

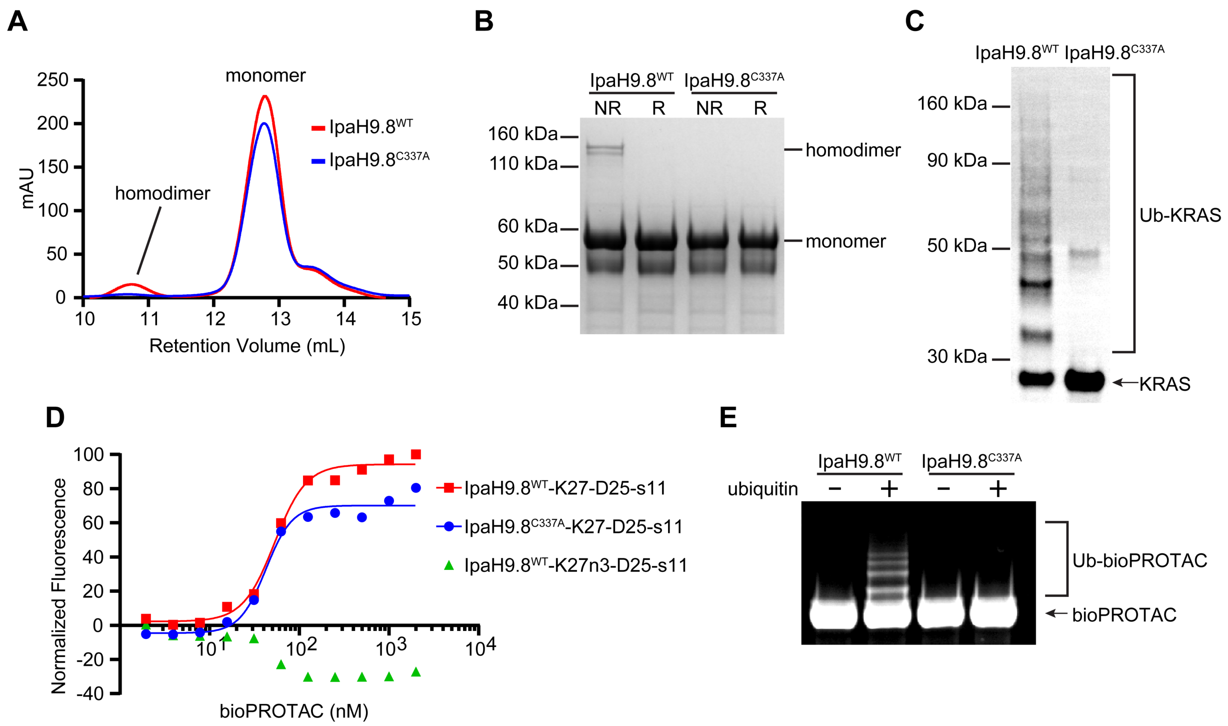

Supplementary Figure 12. Purification and characterization of a catalytically-dead bioPROTAC. **A.** Size exclusion chromatography chromatograms of Ras-targeting bioPROTACs incorporating either the wild-type (WT) IpaH9.8 or an IpaH9.8 with its catalytic cysteine residue mutated to alanine (C337A). **B.** SDS-PAGE analysis of WT or C337A bioPROTACs under non-reducing (NR) or reducing (R) conditions. **C.** *In vitro* KRAS ubiquitination assay using WT or C337A bioPROTACs. **D.** KRAS binding assays were performed with active, catalytically-dead, or non-binding bioPROTACs. Each data point is the mean of n = 2 technical replicates. **E.** Autoubiquitination was assessed *in vitro* with TAMRA-labeled IpaH9.8^{WT} and IpaH9.8^{C337A} bioPROTACs. Source data are provided as a Source Data file.

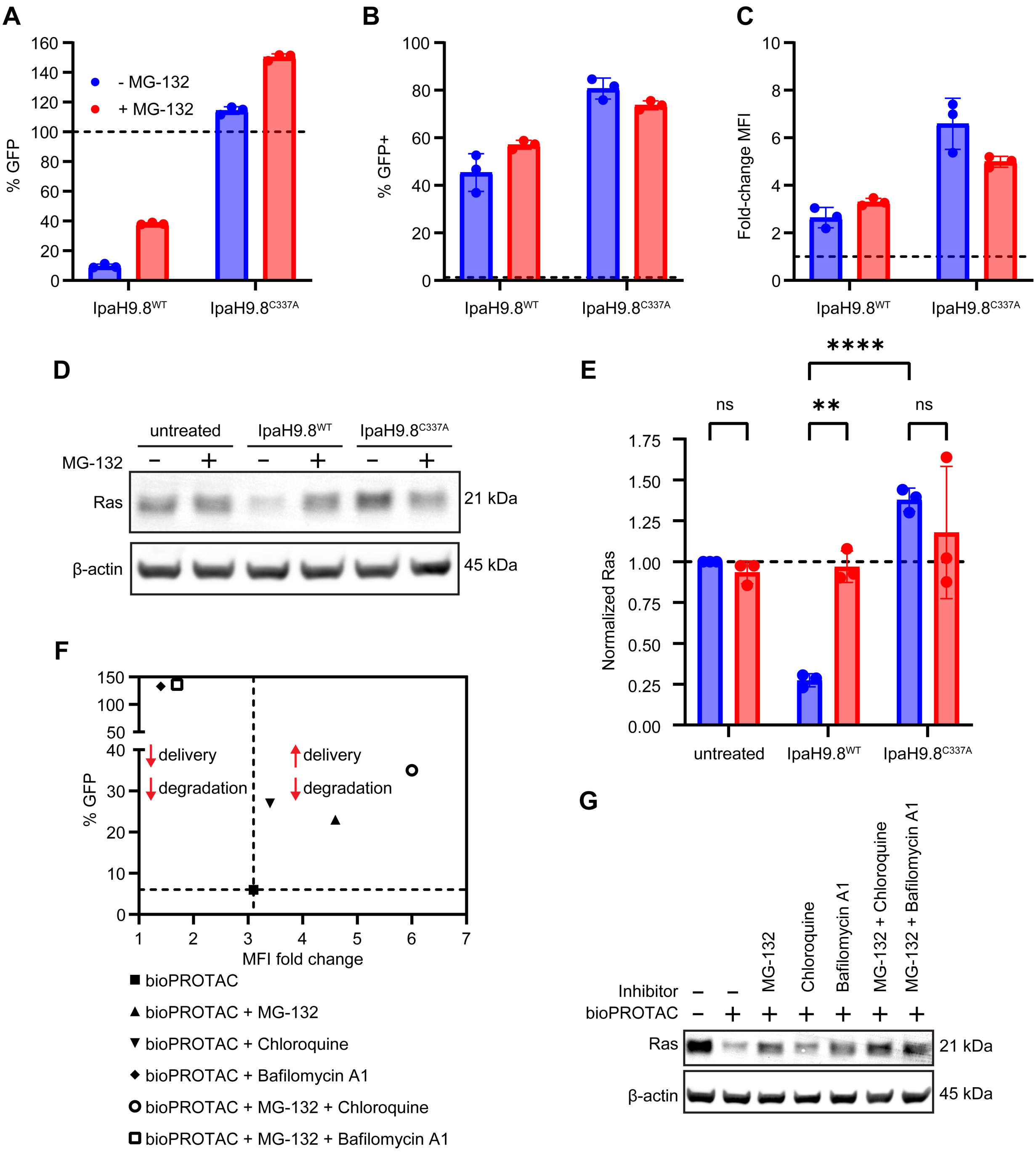

A
B
C
D
E
F
G
- MG-132
+ MG-132
% GFP
% GFP+
Fold-change MFI
IpaH9.8WT
IpaH9.8C337A
untreated
MG-132
Ras
β-actin
21 kDa
45 kDa
Normalized Ras
ns
**
****
delivery
degradation
MFI fold change
bioPROTAC
bioPROTAC + MG-132
bioPROTAC + Chloroquine
bioPROTAC + Bafilomycin A1
bioPROTAC + MG-132 + Chloroquine
bioPROTAC + MG-132 + Bafilomycin A1
Inhibitor
Chloroquine
Bafilomycin A1
MG-132 + Chloroquine
MG-132 + Bafilomycin A1

Supplementary Figure 13. Mechanistic interrogation of Ras bioPROTAC activity. **A.** The degradation activity of K1:bioPROTAC LNPs were assayed by flow cytometry in 293T GFP-KRAS cells. Either WT or C337A IpaH9.8 bioPROTACs were formulated as K1 LNPs and delivered into cells. LNP-treated cells were additionally incubated with or without MG-132 ($n = 3$ technical replicates). **B.** Delivery efficiency represented as percent split GFP complementation in 293T GFP(1-10) cells treated with K1 LNPs encapsulating either active or catalytically-dead bioPROTACs ($n = 3$ technical replicates). **C.** The fold-change MFI in 293T GFP(1-10) cells following treatment with K1:bioPROTAC. **D.** Representative western blot of endogenous Ras in 293T extracts following K1:bioPROTAC treatment with or without MG-132. **E.** Quantitation of samples represented in D. Data were normalized to untreated cells without MG-132 (dotted line). Data are mean ± SD of $n = 3$ biological replicates. Two-way ANOVA followed by multiple comparisons testing was performed. ns $p > 0.05$, ** $p = 0.006$, **** $p < 0.0001$. **F.** Changes to cytosolic delivery in 293T GFP(1-10) cells (x-axis) and target degradation in 293T GFP-KRAS cells (y-axis) following K1:bioPROTAC (IpaH9.8^{WT}-K27-D25-s11) treatment in combination with proteasomal and/or lysosomal inhibitors (10µM MG-132, 50µM chloroquine, 100nM bafilomycin A1). **G.** Endogenous Ras levels in 293T cell lysates following treatment with K1:bioPROTAC with or without proteasomal/lysosomal inhibitors. Treatment time for all panels was 8 hours, and 100nM bioPROTAC was used in protein delivery groups. Source data are provided as a Source Data file.

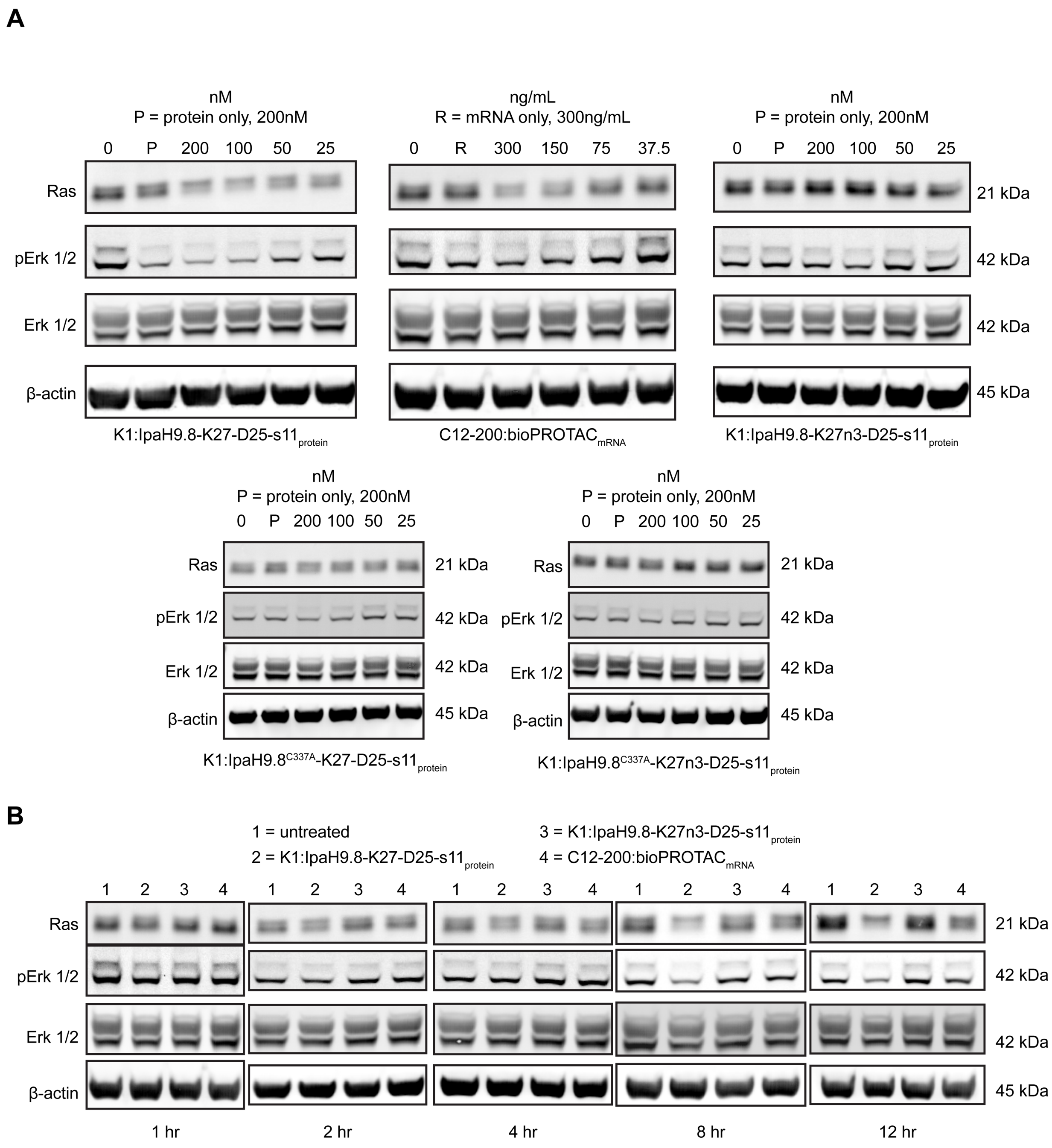

Supplementary Figure 14. Western blot analysis of endogenous protein levels in the MIA PaCa-2 pancreatic cancer cell line following degrader treatment. **A.** Dose-dependent effects of various LNP cargo on endogenous Ras, pErk, and total Erk levels in MIA PaCa-2 following 8-hour treatment and epidermal growth factor stimulation. **B.** Time-course of Ras, pErk, and total Erk levels in MIA PaCa-2 cells treated with degrader-encapsulating LNPs. For protein delivery, the K1 formulation was used, and a dose of 100nM was chosen. For mRNA delivery, C12-200 LNPs were formulated, and a dose of 150ng/mL was chosen. Source data are provided as a Source Data file.

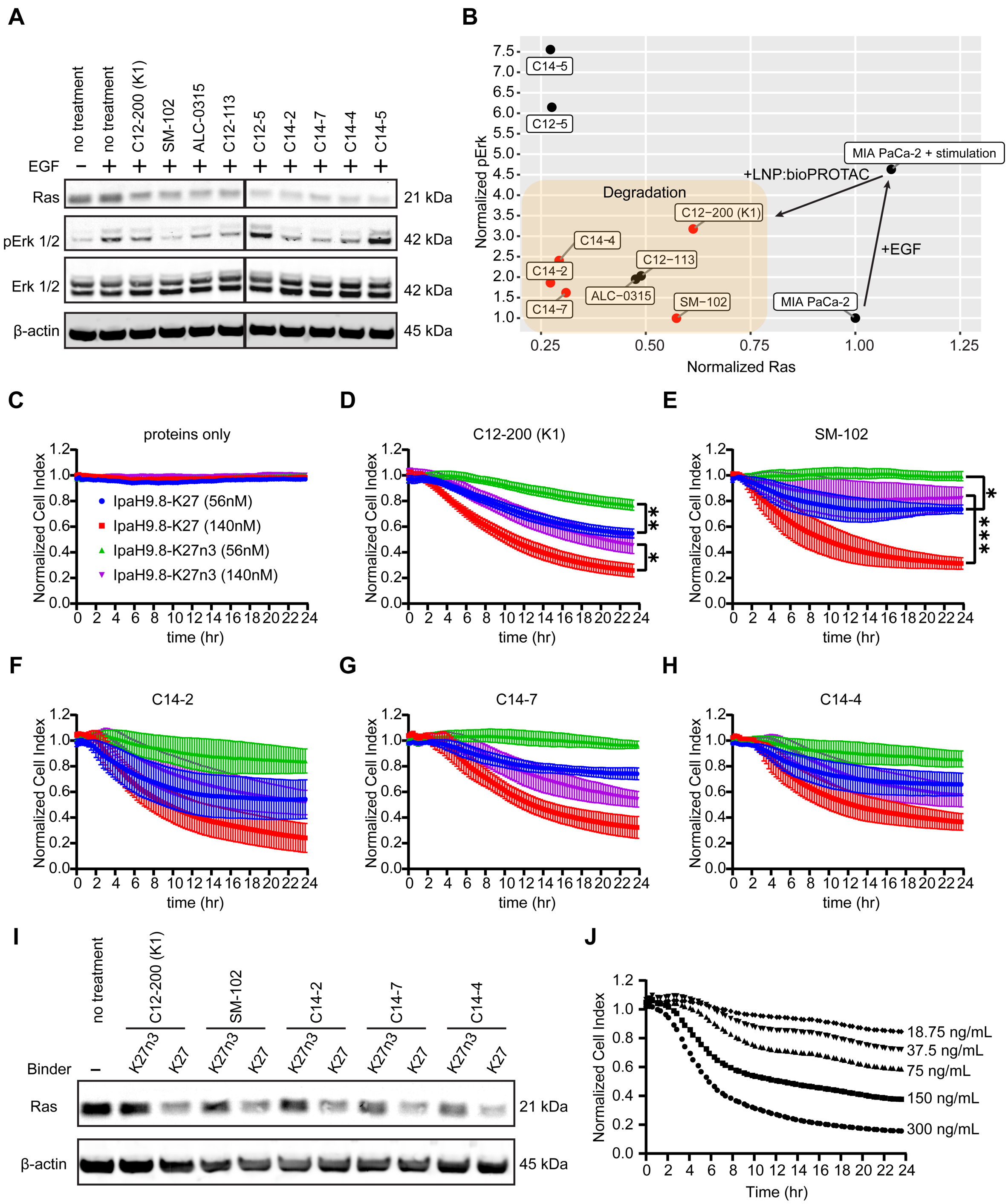

A
no treatment
no treatment
C12-200 (K1)
SM-102
ALC-0315
C12-113
C12-5
C14-2
C14-7
C14-4
C14-5
EGF
Ras
pErk 1/2
Erk 1/2
β-actin
21 kDa
42 kDa
42 kDa
45 kDa
B
Normalized pErk
Normalized Ras
Degradation
C14-5
C12-5
C12-200 (K1)
C14-4
C14-2
C14-7
C12-113
ALC-0315
SM-102
MIA PaCa-2
MIA PaCa-2 + stimulation
+LNP:bioPROTAC
+EGF
C
proteins only
IpaH9.8-K27 (56nM)
IpaH9.8-K27 (140nM)
IpaH9.8-K27n3 (56nM)
IpaH9.8-K27n3 (140nM)
D
C12-200 (K1)
E
SM-102
F
C14-2
G
C14-7
H
C14-4
Normalized Cell Index
time (hr)
I
no treatment
C12-200 (K1)
SM-102
C14-2
C14-7
C14-4
Binder
K27n3
K27
Ras
β-actin
21 kDa
45 kDa
J
Normalized Cell Index
Time (hr)
18.75 ng/mL
37.5 ng/mL
75 ng/mL
150 ng/mL
300 ng/mL

Supplementary Figure 15. Characterization of LNP degraders for Ras-targeted therapy in pancreatic cancer. **A.** In total, 8 ionizable lipids were substituted for the C12-200 lipid used in the base K1 formulation. MIA PaCa-2 cells were treated with 100nM protein LNPs for 8 hours. Cells were stimulated with human epidermal growth factor (EGF) prior to lysis, and extracts were blotted for Ras, pErk and Erk levels. **B.** Both normalized Ras and normalized pErk levels were quantified, and 4 lipids were chosen for further testing (red points). **C.-H.** MIA PaCa-2 were treated with active and null bioPROTACs either as free protein or as LNP:protein formulations. In total, 4 lipids identified from screening were tested, and C12-200 (K1 formulation) was included as a benchmark. Cell proliferation was assayed using the xCELLigence real-time cell analysis system. Data were normalized to untreated controls. For protein-only experiments (C), data points represent the mean of n = 2 biological replicates. In panel D, low dose (blue and green) data points are the mean ± SEM of n = 7 biological replicates, and high dose (purple and red) are mean ± SEM of n = 6 biological replicates. For E-H, data are mean ± SEM of n = 3 biological replicates. For each lipid, two-way ANOVA was performed followed by comparisons between active and null bioPROTACs at matched doses. Only statistically-significant results are indicated. * $p \leq 0.05$, ** $p \leq 0.01$, *** $p \leq 0.001$. **I.** Ras degradation was confirmed for each LNP:protein formulation (100nM) by western blotting. **J.** Normalized proliferation of MIA PaCa-2 cells treated with C12-200 LNPs encapsulating Ras bioPROTAC mRNA. Data are mean of n = 2 biological replicates, with each biological replicate containing 3 technical replicates. Source data are provided as a Source Data file.

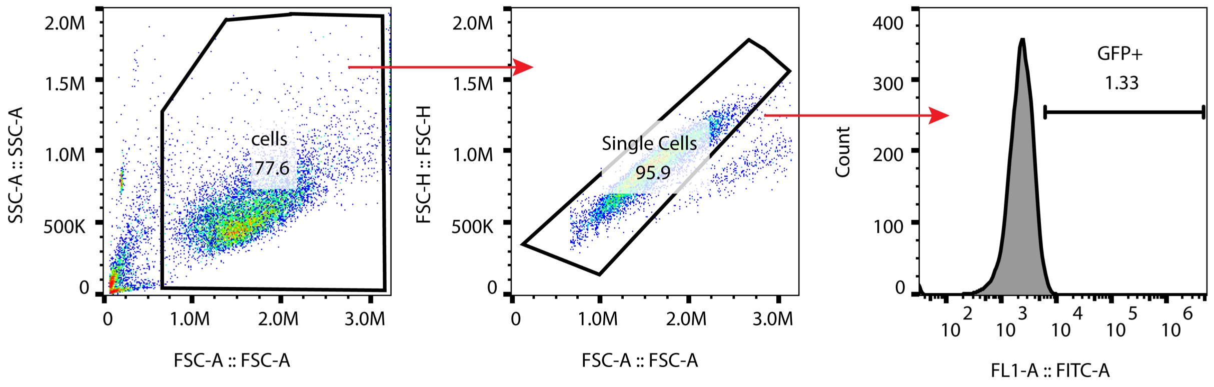

Supplementary Figure 16. Representative gating strategy for flow cytometry experiments. For both degradation experiments and split GFP delivery assays, geometric mean GFP levels were calculated from the entire histogram. For split GFP delivery assays, an additional gate (GFP+ window) was applied, and all cells within this window were considered GFP-positive. This gate was made such that only 1% of the untreated GFP(1-10) cell sample would be considered GFP-positive.

Supplementary Table 1. Initial base LNP formulations for bioPROTAC formulation and delivery.

**Identifier**	K1	C1	B6
**helper lipid type**	DOPE	DSPC	DOPE
**Ionizable lipid type**	C12-200	C12-200	C14-4
**helper lipid (mol %)**	19.0	10.0	10.0
**cholesterol (mol %)**	38.0	38.5	38.5
**C14PEG-2000 (mol %)**	4.0	1.5	1.5
**Ionizable lipid (mol %)**	19.0	25.0	35.0
**DOTAP (mol %)**	20.0	25.0	15.0
**Total (mol %)**	100.0	100.0	100.0

Supplementary Table 2. Library of LNP formulations screened for degradation efficiency.

Identifier	helper lipid type	helper lipid (mol ratio)	cholesterol (mol ratio)	C14PEG-2000 (mol ratio)	C12-200 (mol ratio)	DOTAP (mol ratio)	Total (mol ratio)	helper lipid (mol fraction)	cholesterol (mol fraction)	C14PEG-2000 (mol fraction)	C12-200 (mol fraction)	DOTAP (mol fraction)	Total (mol fraction)
K1	DOPE	15	30	3	15	16	79	0.19	0.38	0.04	0.19	0.20	1.00
D1	DOPE	10	20	2	15	16	63	0.16	0.32	0.03	0.24	0.25	1.00
D3	DOPE	20	40	4	15	16	95	0.21	0.42	0.04	0.16	0.17	1.00
D4	DSPC	10	30	4	15	16	75	0.13	0.40	0.05	0.20	0.21	1.00
D5	DSPC	15	40	2	15	16	88	0.17	0.45	0.02	0.17	0.18	1.00
D6	DSPC	20	20	3	15	16	74	0.27	0.27	0.04	0.20	0.22	1.00
D7	DOPC	10	40	3	15	16	84	0.12	0.48	0.04	0.18	0.19	1.00
D8	DOPC	15	20	4	15	16	70	0.21	0.29	0.06	0.21	0.23	1.00
D9	DOPC	20	30	2	15	16	83	0.24	0.36	0.02	0.18	0.19	1.00

Supplementary Table 3. Physical characterization of select bioPROTAC LNP formulations. Source data are provided as a Source Data file.

LNP	Cargo	Size, average diameter (nm)	PDI	Zeta potential (mV)
C12-200	mRNA	80 ± 1	0.22 ± 0.03	14.5 ± 1.0
K1	protein	167 ± 13	0.41 ± 0.03	6.3 ± 0.5
B6	protein	349 ± 10	0.28 ± 0.05	26.3 ± 3.4
C1	protein	590 ± 55	0.34 ± 0.07	15.2 ± 0.4
D1	protein	202 ± 3	0.17 ± 0.01	18.2 ± 0.2
D3	protein	185 ± 1	0.22 ± 0.02	6.5 ± 3.5

Supplementary References

1. Szklarczyk, D. *et al.* STRING v10: Protein-protein interaction networks, integrated over the tree of life. *Nucleic Acids Res* **43**, (2015).
