# Supplementary material for: Lipid-mediated intracellular delivery of recombinant bioPROTACs for the rapid degradation of undruggable proteins
Source: Nat Commun. 2024 Jul 10;15:5808. doi: 10.1038/s41467-024-50235-x (PMC11237011; doi:10.1038/s41467-024-50235-x)
Supplement: Supplementary file 2 — Reporting Summary [file 41467_2024_50235_MOESM2_ESM.pdf]

nature portfolio

Corresponding author(s): Andrew Tsourkas

Last updated by author(s): Jun 27, 2024

# Reporting Summary

Nature Portfolio wishes to improve the reproducibility of the work that we publish. This form provides structure for consistency and transparency in reporting. For further information on Nature Portfolio policies, see our Editorial Policies and the Editorial Policy Checklist.

## Statistics

For all statistical analyses, confirm that the following items are present in the figure legend, table legend, main text, or Methods section.

| n/a | Confirmed | |
|---|---|---|
| ☐ | ☒ | The exact sample size (*n*) for each experimental group/condition, given as a discrete number and unit of measurement |
| ☐ | ☒ | A statement on whether measurements were taken from distinct samples or whether the same sample was measured repeatedly |
| ☐ | ☒ | The statistical test(s) used AND whether they are one- or two-sided<br>*Only common tests should be described solely by name; describe more complex techniques in the Methods section.* |
| ☒ | ☐ | A description of all covariates tested |
| ☒ | ☐ | A description of any assumptions or corrections, such as tests of normality and adjustment for multiple comparisons |
| ☐ | ☒ | A full description of the statistical parameters including central tendency (e.g. means) or other basic estimates (e.g. regression coefficient) AND variation (e.g. standard deviation) or associated estimates of uncertainty (e.g. confidence intervals) |
| ☐ | ☒ | For null hypothesis testing, the test statistic (e.g. *F*, *t*, *r*) with confidence intervals, effect sizes, degrees of freedom and *P* value noted<br>*Give P values as exact values whenever suitable.* |
| ☒ | ☐ | For Bayesian analysis, information on the choice of priors and Markov chain Monte Carlo settings |
| ☒ | ☐ | For hierarchical and complex designs, identification of the appropriate level for tests and full reporting of outcomes |
| ☒ | ☐ | Estimates of effect sizes (e.g. Cohen's *d*, Pearson's *r*), indicating how they were calculated |

*Our web collection on statistics for biologists contains articles on many of the points above.*

## Software and code

Policy information about availability of computer code

| | |
|---|---|
| Data collection | CytExpert (Agilent) was used for flow cytometry data collection, proteome discoverer for LC-MS/MS |
| Data analysis | graphpad prism v10, flowjo v10, cellprofiler 4.2.6, python 3, FIJI (ImageJ), R |

For manuscripts utilizing custom algorithms or software that are central to the research but not yet described in published literature, software must be made available to editors and reviewers. We strongly encourage code deposition in a community repository (e.g. GitHub). See the Nature Portfolio guidelines for submitting code & software for further information.

## Data

Policy information about availability of data

All manuscripts must include a data availability statement. This statement should provide the following information, where applicable:

- Accession codes, unique identifiers, or web links for publicly available datasets
- A description of any restrictions on data availability
- For clinical datasets or third party data, please ensure that the statement adheres to our policy

Source data are provided with this paper. Proteomics data are available via ProteomeXchange with identifier PXD049388[https://proteomecentral.proteomexchange.org/cgi/GetDataset?ID=PXD049388].

## Research involving human participants, their data, or biological material

Policy information about studies with human participants or human data. See also policy information about sex, gender (identity/presentation), and sexual orientation and race, ethnicity and racism.

| | |
|---|---|
| Reporting on sex and gender | *Use the terms sex (biological attribute) and gender (shaped by social and cultural circumstances) carefully in order to avoid confusing both terms. Indicate if findings apply to only one sex or gender; describe whether sex and gender were considered in study design; whether sex and/or gender was determined based on self-reporting or assigned and methods used. Provide in the source data disaggregated sex and gender data, where this information has been collected, and if consent has been obtained for sharing of individual-level data; provide overall numbers in this Reporting Summary. Please state if this information has not been collected. Report sex- and gender-based analyses where performed, justify reasons for lack of sex- and gender-based analysis.* |
| Reporting on race, ethnicity, or other socially relevant groupings | *Please specify the socially constructed or socially relevant categorization variable(s) used in your manuscript and explain why they were used. Please note that such variables should not be used as proxies for other socially constructed/relevant variables (for example, race or ethnicity should not be used as a proxy for socioeconomic status). Provide clear definitions of the relevant terms used, how they were provided (by the participants/respondents, the researchers, or third parties), and the method(s) used to classify people into the different categories (e.g. self-report, census or administrative data, social media data, etc.) Please provide details about how you controlled for confounding variables in your analyses.* |
| Population characteristics | *Describe the covariate-relevant population characteristics of the human research participants (e.g. age, genotypic information, past and current diagnosis and treatment categories). If you filled out the behavioural & social sciences study design questions and have nothing to add here, write "See above."* |
| Recruitment | *Describe how participants were recruited. Outline any potential self-selection bias or other biases that may be present and how these are likely to impact results.* |
| Ethics oversight | *Identify the organization(s) that approved the study protocol.* |

Note that full information on the approval of the study protocol must also be provided in the manuscript.

# Field-specific reporting

Please select the one below that is the best fit for your research. If you are not sure, read the appropriate sections before making your selection.

☒ Life sciences ☐ Behavioural & social sciences ☐ Ecological, evolutionary & environmental sciences

For a reference copy of the document with all sections, see nature.com/documents/nr-reporting-summary-flat.pdf

# Life sciences study design

All studies must disclose on these points even when the disclosure is negative.

| | |
|---|---|
| Sample size | No sample-size calculation was performed. Sample size for in vitro experiments were chosen between 1-7. Semi-quantitative studies such as western blotting had larger sample sizes (4-5 biological replicates) whereas quantitative studies (flow cytometry) used sample size of 3. biological replicates. Binding and dose response assays were performed as n = 2 or n = 3 technical replicates. These experiments were repeated at least twice with similar results. For the proteomics experiment, a sample size of 1 was chosen. We were not looking to make quantitative judgments between individual proteins. Moreover, our bioPROTACs reliably degraded Ras, so we had high confidence that observed effects were robust. For proliferation studies, 2-7 independent studies (biological replicates) were undertaken for xCelligence studies. We used fewer replicates for the protein-only negative controls (n=2 biological replicates) in order to fit all LNP formulations onto a single E-plate for simultaneous screening. For mRNA proliferation studies, we performed two independent studies with (n=2 biological replicates) and results correlated well between 5 mRNA concentrations. Hypothesis testing was performed on biological replicates and typically included one-way or two-way ANOVA followed by multiple comparisons testing with Bonferroni correction. |
| Data exclusions | For the proteomics study, proteins that were not uniquely identified amongst all treatment groups were excluded. Moreover, a psm filer of 24 was used to generate a high quality data set. This decreased the final protein data set from 4752 to 3827. Western blots that were clearly not transferred properly were excluded. This was determined by excessive bubbles, smears or uneven transfer of a housekeeping protein (tubulin or actin). |
| Replication | multiple batches of proteins and LNPs were used throughout the study. We replicated degradation with protein bioPROTACs throughout the study at various concentrations and treatment times. For viability studies, we replicated results 2 or 3 times on separate days. Western blots were performed on separate days. The proteomics experiment Figure 7) was not repeated. Figure 8D was not repeated, as we validated the result using flow cytometry (8E) under the same conditions. |
| Randomization | this was not relevant to the study. |
| Blinding | this was not relevant to the study. |

# Reporting for specific materials, systems and methods

We require information from authors about some types of materials, experimental systems and methods used in many studies. Here, indicate whether each material, system or method listed is relevant to your study. If you are not sure if a list item applies to your research, read the appropriate section before selecting a response.

## Materials & experimental systems

| n/a | Involved in the study |
|---|---|
| ☐ | ☒ Antibodies |
| ☐ | ☒ Eukaryotic cell lines |
| ☒ | ☐ Palaeontology and archaeology |
| ☒ | ☐ Animals and other organisms |
| ☒ | ☐ Clinical data |
| ☒ | ☐ Dual use research of concern |
| ☒ | ☐ Plants |

## Methods

| n/a | Involved in the study |
|---|---|
| ☒ | ☐ ChIP-seq |
| ☐ | ☒ Flow cytometry |
| ☒ | ☐ MRI-based neuroimaging |

## Antibodies

Antibodies used

primary antibodies:
mouse anti-GFP (RT0265, Bio X Cell; Lebanon, NH, 1:2000), rabbit anti-Ras (3965, Cell Signaling Technology; Danvers, MA, 1:1000), rabbit anti-SAPK/JNK (9252, Cell Signaling Technology, 1:2000), rabbit anti-α-tubulin (2144, Cell Signaling Technology, 1:2000), rabbit anti-Erk1/2 (9102, Cell Signaling Technology, 1:1000), rabbit anti-Bcl-xL (2764, Cell Signaling Technology, 1:1000), and mouse anti-β-actin (3700, Cell Signaling Technology, 1:4000). mouse anti-phospho-Erk1/2 (9106, Cell Signaling Technology, 1:1000).

secondary antibodies:
goat anti-rabbit 680RD (925-68071, LI-COR, 1:15,000), donkey anti-mouse 800CW (925-32212, LI-COR, 1:15,000)

Validation

all CST antibodies were validated by the manufacture with data presented on the website. Other antibodies were previously validated in our lab. For example, we have used the GFP antibody to detect recombinant GFP.

## Eukaryotic cell lines

Policy information about cell lines and Sex and Gender in Research

Cell line source(s)

The following cell lines: 293T, 293T GFP-KRAS, 293T GFP-KRAS/iRFP-CaaX, 293T GFP(1-10), A549, and HT1080 were either obtained from our own stocks or produced for this study. HeLa cells stably expressing HaloTag-GFP-MITO, HaloTag-GFP-SPC25, HaloTag-GFP-MIS12, or HaloTag3x-GFP-TRF1 and U2OS cells stably expressing HaloTag3x-GFP-TRF1 were previously published and generously gifted by Michael Lampson. HCT116 was gifted by Michael Farwell. MIA PaCa-2 was gifted by Gregory Beatty.

Authentication

293T, A549, HCT116, HT1080, MIA PaCa-2 by STR profiling

Mycoplasma contamination

293T, A549, HT1080, HCT116 were thawed from stocks tested negative for mycoplasma

Commonly misidentified lines
(See ICLAC register)

none

## Plants

Seed stocks

*Report on the source of all seed stocks or other plant material used. If applicable, state the seed stock centre and catalogue number. If plant specimens were collected from the field, describe the collection location, date and sampling procedures.*

Novel plant genotypes

*Describe the methods by which all novel plant genotypes were produced. This includes those generated by transgenic approaches, gene editing, chemical/radiation-based mutagenesis and hybridization. For transgenic lines, describe the transformation method, the number of independent lines analyzed and the generation upon which experiments were performed. For gene-edited lines, describe the editor used, the endogenous sequence targeted for editing, the targeting guide RNA sequence (if applicable) and how the editor was applied.*

Authentication

*Describe any authentication procedures for each seed stock used or novel genotype generated. Describe any experiments used to assess the effect of a mutation and, where applicable, how potential secondary effects (e.g. second site T-DNA insertions, mosiacism, off-target gene editing) were examined.*

## Flow Cytometry

### Plots

Confirm that:

- [x] The axis labels state the marker and fluorochrome used (e.g. CD4-FITC).
- [x] The axis scales are clearly visible. Include numbers along axes only for bottom left plot of group (a 'group' is an analysis of identical markers).
- [ ] All plots are contour plots with outliers or pseudocolor plots.
- [ ] A numerical value for number of cells or percentage (with statistics) is provided.

### Methodology

| | |
|---|---|
| Sample preparation | cells were trypsinized, pelleted, and resuspended in FACS buffer (PBS + BSA + EDTA) for analysis |
| Instrument | CytoFLEX V0-B2-R2 flow cytometer (Beckman Coulter). |
| Software | CytExpert software was used for data collection and FlowJo V10 was used for data analysis. |
| Cell population abundance | Typically 10,000 cell events were collected. Following doublet discrimination, 8000-9500 single cells were included in final plots. |
| Gating strategy | cells falling along a the diagonal on a FSC-A vs SSC-A were gated as cells. Doublet discrimination was performed by gating cells along the diagonal on a FSC-A vs FSC-H plot. GFP-positive gates were defined such that only ~1% of the negative control (untreated) cells would fall in that positive gate. |

- [ ] Tick this box to confirm that a figure exemplifying the gating strategy is provided in the Supplementary Information.
